# Trends in Physical Fitness and Nutritional Status among School-Aged Children and Adolescents during the COVID-19 Pandemic in Shaanxi, China—A Cross-Sectional Study

**DOI:** 10.3390/nu14153016

**Published:** 2022-07-22

**Authors:** Zijun Lu, Chuangui Mao, Yuanyuan Tan, Xingyue Zhang, Zheng’ao Li, Ling Zhang, Wenfei Zhu, Yuliang Sun

**Affiliations:** Department of Exercise Science, School of Physical Education, Faculty of Sports and Human Sciences, Shaanxi Normal University, Xi’an 710119, China; luzijun@snnu.edu.cn (Z.L.); maocg117@snnu.edu.cn (C.M.); tanyy98@yeah.net (Y.T.); zhangxingyue@snnu.edu.cn (X.Z.); lizhengao@snnu.edu.cn (Z.L.); zhangling@snnu.edu.cn (L.Z.)

**Keywords:** COVID-19 pandemic, physical fitness, nutritional status

## Abstract

Background: This study aimed to explore the characteristics of trends in physical fitness and nutritional status among school-aged students during the COVID-19 pandemic, which could help to develop targeted guidelines and policies for students (adolescents) to promote health during lockdowns resulting from public health emergencies. Methods: The physical fitness and nutritional status were measured from 2019 to 2021; the correlation between years and grade were analyzed. Results: a declining trend was found in aerobic fitness, strength fitness, speed fitness, and BMI during this lockdown. Conclusion: The changes in dietary behavior and the decrease in outdoor physical activities may be the reason for the decline. Furthermore, differences in subjects’ growth and limited space at home must be considered in the formulation of exercise and nutritional plans. According to the results of our study, exercise for aerobic fitness should receive more attention when students are enduring a pandemic lockdown. In addition, saying “no” to high-calorie foods in the form of snacking and ultra-processed food was the key to improving subjects’ nutritional status.

## 1. Introduction

On 11 March 2020, the World Health Organization labeled COVID-19 as a pandemic. Almost all the countries around the world have suffered and taken measures to limit infection rates, including social distancing, home confinement, stopping sports, closing shops and schools, etc. However, these lockdowns can not only cause psychological problems (anxiety, loneliness, depression, etc.) [1,2] but also affect one’s physical fitness and nutritional status.

Physical fitness, the ability to perform motions or activities, has been widely proved to be significantly associated with health [3]. A low level of physical fitness usually symbolizes a high risk of cardiovascular disease [4], cancer [5], hypertension [6], mental disorders [7], etc. The body mass index (BMI), which is one of the most significant indicators of physical fitness, has been used to measure the nutritional status in many studies [8,9,10]. In addition, nutritional status also plays an important role in health, which means balanced nutrition daily can effectively improve the body’s immunity [11]. Especially during the global pandemic of coronavirus disease 2019 (COVID-19), a better fitness level and immunity not only decreases the chance of mortality but also improves the performance of prognosis after infection [12]. The indirect injury of lockdowns caused by COVID-19 deserves more attention, which reduced people’s physical activities [1,2,13,14], level of physical fitness, and affected their overall health.

The indirect injury of the COVID-19 lockdown affected adolescents and students more than others [2]. Several studies have found that the level of physical fitness of a country’s adolescents is associated with the future profile of the adult population [15,16]. Unfortunately, the fitness level of humans has been decreasing during COVID-19. Theis et al. found that the physical fitness and mental of adolescents with intellectual disabilities decreased significantly from 2019 to July 2020 [17]. Zhou et al. selected 265 adolescents in China and found that their aerobic fitness and speed (explosive force) deteriorated from February 2020 to July 2020 [18]. Sunda et al. observed 48 students who lived normally and 66 students who suffered from the pandemic lockdown in Croatia. The results showed that the 66 students’ strength fitness decreased [19].

On the other hand, the COVID-19 lockdown also had an effect on the nutritional status of humans. Such as a change in dietary behavior was associated with increased snacking [20] and a higher consumption of ultra-processed food [21]. Legg et al. found that the obesity (BMI > 30) prevalence of male adults increased from 2018 to 2021 [22]. A Korean study including 139 children aged 6–12 years [23] and a German study that included 150,000 children [24] found that the BMI Z-score increased significantly over the year, especially during the first three months of 2020.

Some studies examined the impact of the COVID-19 pandemic on students’ physical fitness and nutritional status [18,25]. However, to our knowledge, there is little work that has investigated the trends in a large population-based cohort of students from 6 to 24 years old before and during the COVID-19 pandemic.

Therefore, this study aimed to explore the characteristics of trends in physical fitness and nutritional status among school-aged children and adolescents during the COVID-19 pandemic, in which the physical fitness and nutritional status were measured before and during COVID-19, from 2019 to 2021. We hypothesized that (1) the BMI of students increased during the COVID-19 lockdown. (2) The performance of aerobic fitness and strengthen fitness decreased during the COVID-19 lockdown.

## 2. Methods

### 2.1. Participants

**Figure 1:**
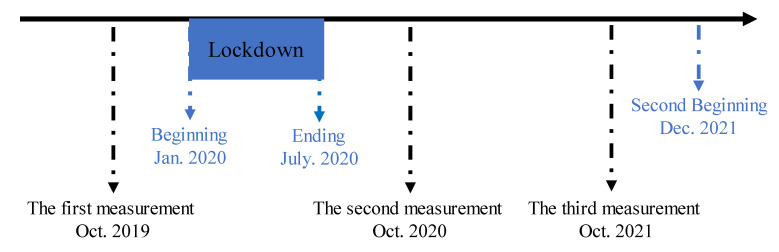
The timeline of COVID-19 pandemic in Shaanxi and the time of measurement.

Overall, 27,181 students aged 6–22 years from primary school, middle school, high school, and college took the physical fitness test, which has been taken for three years (2019–2021) and is always completed during the fall semester (September–February) (Figure 1). Based on the Chinese National Student Physical Fitness Standard (CNSPFS), the participants were randomly selected in each grade. In addition, “sports specialists” in primary school, middle school, and high school were excluded from participation as well as students from the school of physical education in college. In addition, female students during menstruation and participants with a history of injury were also excluded. All evaluators were given a testing manual and two training seminars to reduce errors during the test (Table 1). The experimental procedure was approved by a local ethics committee (no. 202016001 2020-09).

### 2.2. Assessments

The Chinese National Student Physical Fitness Standard (CNSPFS) was applied to assess the physical fitness of students, which has been considered the largest nationally representative survey of the health status of Chinese children and adolescents [26]. Participants’ performance in eight indicators of physical fitness status (50 m running, standing long jump, sit and reach, 800/1000 m running, sit-up, chin-up, 50 m × 8, jumping rope) were classified into five fitness batteries, including strength, endurance, flexibility, speed, and coordination [27]. In addition, the indicators of nutritional status (body mass index) and vital capacity [3] were measured in our study (Table 2).

#### 2.2.1. Body Mass Index (BMI)

BMI was a significant factor used to assess body composition, [28] which was calculated as weight in kilograms divided by squared height in meters (kg/m^2^). Weight was measured as subjects were barefoot and standing naturally in the center of a scale, keeping their body steady. The results were in kilograms and accurate to one decimal place. When measuring height, the subjects stood barefoot, back to the column with their torso naturally straight, head upright, and eyes looking straight ahead. The results were in centimeters and accurate to one decimal place. According to the standard of WHO, the cut-offs included Overweight, Obesity, Medium, Thinness, and Severe Thinness [29]. Overweight and Obesity were combined as overnutrition. Thinness and Severe thinness were combined as undernutrition. All the participants received BMI assessment.

#### 2.2.2. Vital Capacity (VC)

Vital capacity (VC) refers to the maximal volume of air that can be expired following maximum inspiration. It is the total of tidal volume (V), inspiratory reserve volume (IRV), and expiratory reserve volume (ERV) (VC = V + IRV + ERV) [30]. During the test, the subjects should keep standing, trying their best to inhale, aim their mouth at the equipment’s blowing nozzle, and exhale until they could not. This test was performed twice, with the maximum value as the result. All the participants need to receive the vital capacity assessment.

#### 2.2.3. Strength

Strength tests in our study included arm and shoulder strength (chin-up), lower limb strength (standing long jump), and abdomen strength (sit-up). The chin-up test was required for the male students aged 13–22 years, the sit-up test was required for female students aged 13–22 years, and all the students aged 13–22 years need to perform the standing long jump test. Standing long jump test requires the subjects to keep their legs naturally apart, jumping with both legs in place. The evaluators should measure the distance from the trailing edge of the jump wire to the nearest landing spot. While performing the chin-up test, the subjects should jump up to hold the horizontal bar with both hands in an upright grip, keeping the body in a straight-armed hanging position. One standard chin-up required their lower jaw to exceed the upper edge of the horizontal bar and restore the straight-arm hanging position. The Sit-up test required the subjects continuous one movement (holding the head with both hands and tucking the abdomen to sit up) for 1 min. In addition, the evaluators should keep the subjects’ knee flexion at 90 degrees.

#### 2.2.4. Endurance

Endurance tests included 50 m × 8 and 800/1000 m running. The students aged 10–12 years need to take 50 m × 8, the male students aged 13–22 need to take 1000 m running, and the female students aged 13–22 need to take 800 m running. All the results should be accurate to 1 decimal place. The evaluators should organize students to warm up before the test and relax after the test.

#### 2.2.5. Flexibility

Sit and reach were performed to test the flexibility of the students. During the test, the subjects should put both hands together with the palms down, keeping the knees straight, then bend the upper body forward, and try their best to use the finger to push the vernier smoothly forward. The results were in centimeters and accurate to 1 decimal place. The evaluators should organize the students to stretch before the test. This test was required for all the subjects aged 6–22 years.

#### 2.2.6. Speed

50 m running was performed for all students aged 6–22 years to test the speed. The evaluators should ensure at least 2 students in a group and all the subjects must use the standing start. The results were in seconds and accurate to 1 decimal place. The evaluators should organize the students to warm up before the test.

### 2.3. Statistical Analysis

The Statistical Package for the Social Science (SPSS 26.0) was used for the data analysis. The data of CNSPFS were checked for Gaussian distributions using k-density plots and the extreme outliers were removed using a z-score cut-point of ±3.0. The one variable analysis in the general linear model (GLM) was used to measure the association between physical fitness & nutritional status and years. The difference in gender (male/female) and region (urban/rural) were calculated using independent *t*-tests. Age was entered as a covariate within each of the models.

## 3. Results

Almost all indicators were affected by the years (COVID-19 Pandemic) except the grade of sit and reach in college.

### 3.1. Trends in Nutritional and Growth Status and Visual Acuity

In terms of growth and nutritional status, there was a decrease in severe thinness and thinness and the group of Medium remained stable basically. However, the increase in overweight and obesity was obvious (Table 3).

**Figure 2:**
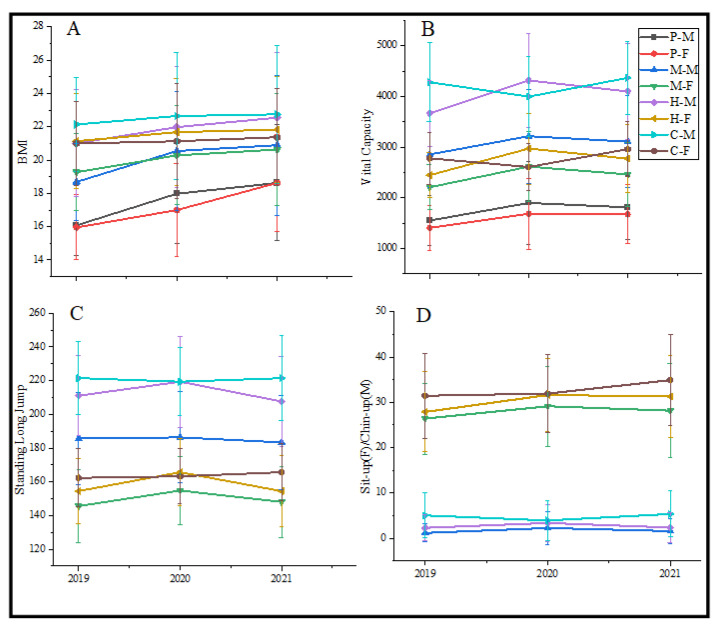
The trend of BMI, vital capacity, standing long jump, sit-up, and chin-up. (**A**) The trend of BMI; (**B**) The trend of vital capacity; (**C**) The trend of standing long jump; (**D**) The trend of sit up and chin up. P: Primary school; M: Middle school; H: High school; C: College; M: Male; F: Female.

The BMI of students in primary school, middle school, and high school increased from 2019 to 2021. However, this trend flattened in college. The increase between primary school and middle school was more significant than in other growth phases (Figure 2A).

The trend of vital capacity deserves to be mentioned, which decreased from 2019 to 2020, while increased from 2020 to 2021 in primary school, middle school, and high school. On the contrary, the one of the college increased from 2019 to 2020 and decreased from 2020 to 2021 (Figure 2B).

### 3.2. Trends in Physical Fitness

The distance of standing long jump increased from 2019 to 2020 and decreased from 2020 to 2021 in the high school students and female middle school students (Figure 2C). There was no significant difference in the grades of Chin-up and Sit-up (Figure 2D). It deserves to be mentioned that the passing rate of the Chin-up was low (2019: 8.36%, 2020: 12.58%, 2021: 11.36%), which means the upper strength of male students needs to be improved in the future.

**Figure 3:**
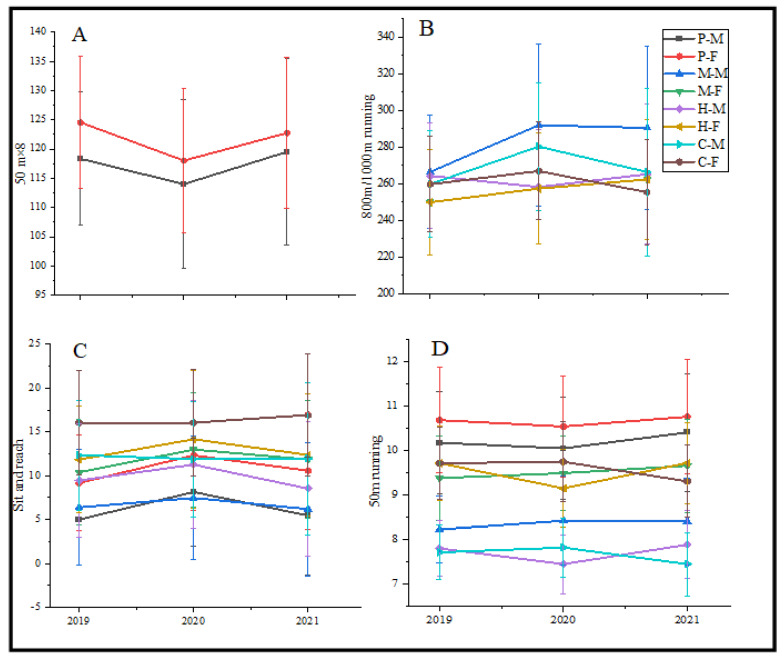
The trend of 50 m × 8, 800/1000 m running, Sit and reach, 50 m running. (**A**) The trend of 50 m × 8; (**B**) The trend of 800 m/1000 m; (**C**) The trend of sit and reach; (**D**) The trend of 50 m run-ning. P: Primary school; M: Middle school; H: High school; C: College; M: Male; F: Female.

The grade of 800 m running keeps decreasing from 2019 to 2021 in female middle school and high school students. The grade of 800/1000 m running of students in college and male students in middle school both exhibited the same trend, which decreased from 2019 to 2020 and increased from 2020 to 2021 (Figure 3A). On the contrary, the grade of 1000 m running in male high school students increased from 2019 to 2020 and decreased from 2020 to 2021, which was the same as the grade of 50 m × 8 in primary school students (Figure 3B).

The grade of sit and reach in primary school, middle school, and high school students exhibited the same trend, which increased from 2019 to 2020 and decreased from 2020 to 2021 (Figure 3C). The grade of 50 m running increased from 2019 to 2020 and decreased from 2020 to 2021 in primary school and college students. On the contrary, the grade of high school students decreased from 2019 to 2020 and increased from 2020 to 2021 (Figure 3D).

### 3.3. Difference in Gender and Region

As summarized in Table 4, the indicators almost showed a significant gender difference (*p* < 0.01), except for the BMI of students in middle school and visual acuity of students in college.

The difference in the region could also be found in most indicators (Table 5), except for the BMI, visual acuity (left), vital capacity, the grades of 800/1000 m running, and 50 m running of students in middle school, the vital capacity and the grades of 800/1000 m running of students in high school, the quantity of jumping rope of students in primary school. Additionally, we also found a correlation between the region and BMI in Primary school (*p* < 0.05) and high school students (*p* < 0.01).

## 4. Discussion

This cross-sectional study evaluated the trend in physical fitness and nutritional status (BMI) of school-aged children and adolescents during the COVID-19 pandemic lockdown. Overall, a declining trend was found in aerobic fitness, strength fitness, speed fitness, and BMI during this lockdown. The results contribute to developing a targeted intervention strategy for the promotion of physical fitness and nutritional status during the lockdown caused by the public health emergency.

In our study, we observed a decrease in severe thinness and thinness as well as an increase in overweight and obesity. The changes in dietary behavior may be the reason for this trend. Adolescents and children usually consumed more snacking and ultra-processed food during the lockdown [20,21]. Additionally, the decrease in outdoor physical activities may also be the other reason, although there were many studies have focused on the contributions of indoor physical activities during the pandemic [1,31,32], the significant decrease in physical activity equivalent (PAE) has been the main reason for the increasing BMI [1,2,13,14].

However, this continued increasing trend was not entirely consistent with the timeline of the pandemic lockdown, which means the lockdown may not the only reason for the increase in BMI. Along with the success of poverty eradication, a convergence of urban and rural lifestyles has been occurring in China, resulting in similar changes in nutritional status across both settings as urbanization advanced [33]. This process of urbanization was usually accompanied by the improvement of foods (a shifting nutritional environment with consumption of high-calorie foods) and lifestyles (lower levels of work-related physical activities). This trend not only occurred in China but also in most low-and-middle-income regions [34]. Although there was a difference between rural and urban students in primary school and high school in our study, the increasing rate of BMI was the same or even faster.

This association has been proved by many studies, that high BMI, which means overweight and obesity, usually affects the grades of endurance running [35,36]. The increasing BMI also results in a decrease in the performance of aerobic fitness in our study. Especially from 2019 to 2020, almost all the students’ grades in endurance running decreased except for the male students in high school and primary school. Although there was a rebound from 2020 to 2021, the grade was barely back to the original.

According to the special trend of male students in high school, the different lengths of lockdown and gender may be the reason. To ensure the quality of education, the high schools in some areas where the pandemic was not so serious may resume the classes, during which the school usually organized students to jog regularly. Moreover, the male students’ physical activities were more than female students in high school [37]. To summarize, regular jogging and more activities explain the difference in male students in high school. In addition, the difference in growth may be the reason for the special trend in primary school students. Growth was the main indicator that affect the physical fitness level of primary school students, which means the pandemic lockdown’s effect was covered by the growth.

On the contrary, the effect of pandemic lockdown on middle school students’ grades in endurance running was more significant than in other groups, the inclusion of physical fitness tests in the final exam may explain this difference. To promote the grades of students’ physical fitness, middle schools always organized students to practice regularly, while pandemic lockdown stops the practice, leading to a substantial decline.

Interestingly, the performance of speed and lower limb strength fitness (50 m running and sitting long jump) improved from 2019 to 2020 in high school students. To the same, the performance of flexibility fitness (sit and reach) and vital capacity also increased during the pandemic lockdown. These results were not only found in our study [18,19]. The limited exercise space at home may contribute to fewer exercise patterns to choose from while stretching and resistance training were convenient to proceed with. On the other hand, this suggests that the increase in adolescents was continuous, the pandemic lockdown may only affect the growth of various physical fitness indicators to various degrees. Nonetheless, the principles of the underlying physiological mechanism deserve to be further explored in the future.

The main strengths of this study are (1) the time of the measurement included the two phases of the COVID-19 pandemic lockdown, which could explain the mechanism of physical fitness and nutritional status in the effect of pandemic lockdown and the recovery after the pandemic lockdown. (2) the subjects in the study contain the students from 6–22 years, which could combine the growth and pandemic lockdown to explain the change in physical fitness and nutritional status. To our knowledge, this is the first study that selected the students from primary school to college during the COVID-19 pandemic lockdown.

Some limitations of this study include: (1) The subjects were not the same from 2019 to 2021, therefore this is only a cross-sectional study. The effect of individual differences on the results deserves to be considered. (2) BMI is a significant indicator to evaluate the physical fitness and nutritional status of human. However, it could be affected by Body composition. The future studies could use more accurate methods such as the bioimpedance or Dual Energy X-ray Absorptiometry (DEXA).

## 5. Conclusions

Findings from this cross-sectional study indicate that the nutritional status (BMI) and aerobic fitness of students in Shaanxi, China both decreased during the COVID-19 pandemic lockdown. Although there was a rebound from 2020 to 2021, the grade was barely back to the original. The changes in dietary behavior and the decrease in outdoor physical activities may be the reason.

Although the pandemic lockdown has effectively curbed the spread of the COVID-19 pandemic, it also indirectly led to the decline in physical fitness and nutritional status of students aged from 6 to 22 years. Unfortunately, the pandemic is continuing, and many regions around the world are suffering currently. Therefore, developing targeted guidelines and policies for students (adolescents) to promote health during the lockdown resulting from public health emergencies is essential.

Furthermore, the difference in growth and limited space at home must be considered in the formulation of exercise and nutritional plans. According to the results of our study, exercise for aerobic fitness should be paid more attention to while the students suffer from the pandemic lockdown. In addition, saying “no” to high-calorie foods like snacking and ultra-processed food was the key to improving the nutritional status.

## Figures and Tables

**Table 1:** Sample characteristics (n = 27,181).

			Primary School	Middle School	High School	College		Total	
Male	Urban	2019	693	432	597	2019	730	12,094	27,181
		2020	955	476	433				
		2021	813	404	408	2020	451		
	Rural	2019	755	451	582				
		2020	953	477	446	2021	412		
		2021	816	404	406				
Female	Urban	2019	832	470	695	2019	1534	15,087	
		2020	954	474	473				
		2021	814	409	800	2020	900		
	Rural	2019	875	503	620				
		2020	946	468	469	2021	828		
		2021	804	405	814				

**Table 2:** Test projects and test groups.

Test	6–12 Years	10–12 Years	13–22 Years
Height/Weight	●		●
Vital capacity	●		●
Visual acuity	●		●
Sit-up (Female)			●
Chin-up (male)			●
Standing long jump			●
50 m × 8		●	
800 m running (female)			●
1000 m running (male)			●
Sit and reach	●		●
50 m running	●		●

The “●” represents the test items that different participants received.

**Table 3:** The nutritional status of Chinese children and adolescents aged 7–22 years old in the CNSSCH, from 2019 to 2021.

		2019	2020	2021
Sample size		9769	8875	8537
Nutritional status	Gender			
Severe thinness	Male	213 (5.03%)	112 (2.66%)	48 (1.31%)
	Female	137 (2.48%)	79 (1.69%)	39 (0.79%)
Thinness	Male	822 (19.4%)	393 (9.37%)	274 (7.49%)
	Female	973 (17.6%)	530 (11.31%)	402 (8.24%)
Medium	Male	2641 (62.3%)	2345 (55.95%)	1941 (52.99%)
	Female	3965 (71.71%)	3169 (67.66%)	3222 (66.11%)
Overweight	Male	528 (12.46%)	891 (21.26%)	850 (23.22%)
	Female	428 (7.74%)	718 (15.32%)	950 (19.49%)
Obesity	Male	34 (0.81%)	451 (10.75%)	549 (14.98%)
	Female	26 (0.46%)	188 (4.02%)	262 (5.37%)

**Table 4:** The difference in the performance of physical fitness in gender (male = 12,094, female = 15,087).

	Primary School		Middle School		High School		College	
	T	P	T	P	T	P	T	P
BMI	10.705	<0.01	0.077	0.938	2.996	0.003	11.559	<0.01
Vital Capacity	13.41	<0.01	31.372	<0.01	64.893	<0.01	61.679	<0.01
Standing Long Jump	-	-	53.004	<0.01	89.57	<0.01	82.312	<0.01
Sit-up	12.225	<0.01	-	-	-	-	-	-
Chin-up	-	-	-	-	-	-	-	-
Sit and reach	−35.947	<0.01	−26.983	<0.01	−15.846	<0.01	−17.631	<0.01
50 m × 8	−10.628	<0.01	-	-	-	-	-	-
Jumping rope	−9.195	<0.01	-	-	-	-	-	-
50 m running	−18.865	<0.01	−45.074	<0.01	−86.846	<0.01	−71.739	<0.01
800 running	-	-	-	-	-	-	-	-
1000 m running	-	-	-	-	-	-	-	-

**Table 5:** The difference in the performance of physical fitness in region (urban = 11,132, rural = 11,194).

	Primary School		Middle School		High School		College	
	T	P	T	P	T	P	T	P
BMI	4.83	<0.01	0.764	0.445	2.807	0.037	4.83	<0.01
Vital Capacity	12.605	<0.01	0.03	0.976	−0.905	0.366	12.605	<0.01
Standing Long Jump	-	-	−5.132	<0.01	−2.956	0.003	-	-
Sit-up	0.638	<0.01	−6.293	<0.01	12.654	<0.01	0.638	<0.01
Chin-up	-	-	−5.665	<0.01	−4.648	<0.01	-	-
Sit and reach	−4.886	<0.01	−3.928	<0.01	−3.76	<0.01	−4.886	<0.01
50 m × 8	6.072	<0.01	-	-	-	-	6.072	<0.01
Jumping rope	−1.226	0.220	-	-	-	-	−1.226	0.220
50 m running	4.007	<0.01	−0.375	0.708	−2.065	0.039	4.007	<0.01
800 running	-	-	0.788	0.431	−0.443	0.658	-	-
1000 m running	-	-	0.205	0.838	−0.452	0.651	-	-

## Data Availability

Not applicable.
